# Metabolic reprogramming in sepsis-associated encephalopathy: emerging mechanisms, candidate biomarkers, and future therapeutic directions

**DOI:** 10.3389/fmed.2026.1821690

**Published:** 2026-05-14

**Authors:** Mei Huang, Weiwei Qian, Daxiu Lin, Yihua Zeng, Yan Zhou, Shurong Zhang, Yaling Luo, Zhi Li, Xiaodong Du

**Affiliations:** 1 Department of Critical Care Medicine, West China Hospital/West China School of Nursing, Sichuan University, Chengdu, China; 2 Department of Emergency Medicine, West China Hospital, Sichuan University, Chengdu, China; 3 Department of Pulmonary and Critical Care Medicine, West China Hospital/West China School of Nursing, Sichuan University, Chengdu, China; 4 Department of Critical Medicine, Chengdu Shangjin Nanfu Hospital, Chengdu, China

**Keywords:** biomarkers, immunometabolism, metabolic reprogramming, neuroinflammation, precision medicine, sepsis-associated encephalopathy

## Abstract

Sepsis-associated encephalopathy (SAE) affects up to 70% of septic patients, causing significant mortality and long-term cognitive impairment. Traditional mechanisms–neuroinflammation, blood-brain barrier disruption, and oxidative stress–fail to explain SAE heterogeneity and post-septic cognitive sequelae. Metabolic reprogramming, the systematic remodeling of cellular metabolic pathways, has emerged as an integrative paradigm for understanding SAE pathogenesis. This review examines how metabolic reprogramming drives SAE through four core pathways: glycolysis/Warburg effect, tricarboxylic acid cycle dysfunction, lipid dysregulation, and amino acid disturbance. We discuss cell-type-specific adaptations in microglia, neurons, and astrocytes, and the modulatory role of gut-brain axis crosstalk. We further evaluate metabolomics-driven biomarkers for early diagnosis and emerging metabolic-targeted therapies, including metformin and dichloroacetate. Finally, we highlight single-cell metabolomics and multi-omics integration as critical frontiers toward precision medicine for SAE.

## 1 Introduction

Sepsis, defined as life-threatening organ dysfunction caused by a dysregulated host response to infection, constitutes the leading cause of morbidity and mortality among patients in intensive care units (ICUs) worldwide. As its most frequent neurological complication, sepsis-associated encephalopathy (SAE) carries a particularly heavy clinical burden. The diagnosis of SAE is primarily based on the presence of acute brain dysfunction in septic patients, with exclusion of direct central nervous system infection, structural lesions, or other identifiable causes (1, 2).

Epidemiological data reveal an alarming incidence of SAE, with reported rates among ICU septic patients varying widely, estimated between 9 and 71% (3, 4). This substantial variability stems primarily from heterogeneous diagnostic criteria, diverse study populations, and differences in monitoring sensitivity, yet also reflects the ubiquity of SAE from another perspective. The emergence of SAE serves as a powerful predictor of poor prognosis and is closely associated with significantly increased patient mortality. Some studies report that in-hospital mortality among septic patients with SAE may reach 20%−30%, and even higher in certain critically ill subgroups (5–9). However, the threat of SAE extends far beyond the patient's discharge from the ICU. Accumulating evidence indicates that sepsis survivors, particularly those who experienced SAE, face long-term cognitive dysfunction. These sequelae manifest as memory impairment, attention deficits, and executive dysfunction, severely compromising patients' quality of life, social functioning, and capacity for independent living, while increasing their future risk of dementia (10–14). Estimates suggest that up to 30% or more of severe sepsis survivors experience cognitive impairments persisting for months to years. This progression from acute brain dysfunction to chronic neurodegenerative changes constitutes one of the most formidable challenges in SAE research, representing a core component of “Post-Sepsis Syndrome” (PSS) (15–18).

Over the past decades, exploration of SAE pathophysiology has achieved considerable advances, establishing several classical theoretical frameworks: (1) Neuroinflammation: Systemic inflammatory storms penetrate the central nervous system (CNS) through multiple pathways (e.g., circulating inflammatory cytokines, activated immune cells), activating microglia and astrocytes to release abundant pro-inflammatory mediators, resulting in neuronal injury (19); (2) Blood-brain barrier (BBB) disruption: Inflammatory mediators and endothelial cell damage increase BBB permeability, allowing harmful substances, plasma proteins, and peripheral immune cells to enter the brain parenchyma, compromising the immune-privileged status of the CNS (20); (3) Mitochondrial damage and oxidative stress: Systemic hypoxia, hypoperfusion, and inflammatory responses during sepsis directly damage neuronal and glial mitochondria, leading to impaired energy production like ATP (adenosine triphosphate) depletion and excessive generation of reactive oxygen species (ROS), triggering oxidative stress and apoptosis (21); (4) Additional mechanisms: Including excitatory amino acid toxicity, neurotransmitter imbalance, and cerebral blood flow dysregulation (22).

Although these mechanisms are crucial for explaining certain pathological features of SAE, they have often been investigated as independent, parallel pathways, fostering a “fragmented” understanding. This perspective fails to address several critical questions: Why do similar levels of systemic inflammation result in markedly different neurological outcomes? How do cells, particularly immune and neural cells, make adaptive or maladaptive functional choices within harsh microenvironments? How do acute-phase inflammation and energy failure translate into long-term structural and functional remodeling? These limitations underscore the urgent need to identify an upstream core mechanism capable of integrating these diverse pathological processes and explaining their intrinsic drivers.

The concept of “metabolic reprogramming,” initially widely recognized in tumor biology, describes the systematic remodeling of core metabolic pathways (e.g., glucose, lipid, and amino acid metabolism) that cells undergo to adapt to functional demands such as proliferation and invasion (23). In recent years, this concept has been successfully introduced into immunology, establishing the frontier discipline of “immunometabolism,” which reveals the tight coupling between immune cell activation, differentiation, and function with their metabolic states (24, 25).

In the context of sepsis, metabolic reprogramming is no longer confined to peripheral immune cells but is regarded as a universal, systemic phenomenon involving multiple organs and cell types (26–28). For SAE specifically, metabolic reprogramming serves as the central nexus connecting neuroinflammation, BBB disruption, mitochondrial damage, and neuronal dysfunction. It represents not merely a passive cellular response to stressors such as inflammation and hypoxia, but rather an active regulatory process determining cellular phenotype and fate (19, 29). For instance, pro-inflammatory (M1) polarization of microglia depends on metabolic shifts toward glycolysis (the Warburg effect), whereas the survival or death fate of neurons during energy supply disruption depends directly on their metabolic flexibility (30–32).

Therefore, adopting “metabolic reprogramming” as a core framework for studying SAE may enable the unification of neuroinflammation (immune cell metabolism), energy crisis (mitochondrial function), and cellular damage (neuronal/glial metabolism) within a single logical framework. This approach aims to illuminate the dynamic evolution of cellular function and metabolic states from the acute to chronic phases of disease, potentially facilitating explanation of long-term cognitive impairment formation. Key enzymes and pathways within metabolic networks may provide abundant resources for developing novel diagnostic biomarkers and therapeutic targets, though their clinical utility remains to be validated.

While recent reviews have comprehensively addressed neuroinflammation, blood-brain barrier disruption, and gut-brain axis interactions in SAE (19, 33, 34), a systematic synthesis centered specifically on metabolic reprogramming as an integrative framework remains limited. This review fills this gap by: (1) dissecting cell-type-specific metabolic adaptations across the neurovascular unit; (2) connecting metabolic biomarker discovery with therapeutic targeting strategies; and (3) critically evaluating the translational potential while acknowledging current evidence limitations.

Throughout this review, we employ an evidence-grading framework to distinguish the strength and source of supporting data: [H] indicates human clinical or postmortem studies; [A] indicates animal model studies; [I] indicates *in vitro* or cellular studies; and [E] indicates evidence extrapolated from related disease contexts (e.g., cancer immunometabolism, neurodegenerative diseases) that requires direct validation in SAE. This framework is essential given that some mechanistic insights discussed herein are derived from broader immunometabolism literature and may not yet be fully established in SAE specifically.

## 2 SAE and metabolic reprogramming

The influence of metabolic reprogramming in SAE is profound and multifaceted, manifesting not only as global remodeling of core metabolic pathways, but also as specific metabolic phenotype alterations in key brain cell types, while simultaneously being modulated by metabolic crosstalk from distal organs, particularly the intestine.

### 2.1 Systemic remodeling of four core metabolic pathways

The systemic stress response elicited by sepsis profoundly perturbs metabolic homeostasis within the central nervous system, triggering dramatic fluctuations in cerebral metabolic networks. Through metabolomics technologies, researchers have observed extensive alterations in metabolite profiles across brain tissue, cerebrospinal fluid, and even peripheral blood samples derived from both animal models and patients with SAE (35–37). These alterations do not occur in isolation but rather center on the systemic remodeling of several core metabolic pathways.

#### 2.1.1 Enhanced glycolysis and the emergence of the Warburg effect

Enhanced glycolysis, particularly the emergence of the Warburg effect, represents one of the most prominent features of early metabolic reprogramming in SAE (38, 39). Under physiological conditions, cerebral neurons predominantly rely on mitochondrial oxidative phosphorylation to efficiently generate ATP to meet their substantial energy demands (40). However, within the pathological microenvironment of SAE, multiple brain cell types—especially microglia and infiltrating immune cells—undergo metabolic shifts from oxidative phosphorylation toward aerobic glycolysis, manifesting the Warburg effect (19). This process is driven by key signaling pathways including hypoxia-inducible factor-1α (HIF-1α) and mammalian target of rapamycin (mTOR). HIF-1α forcibly diverts glucose metabolism toward lactate production through upregulation of glucose transporters and key glycolytic enzymes, even when oxygen supply remains adequate. This metabolic conversion carries profound pathophysiological implications (41, 42). On one hand, enhanced glycolysis rapidly generates ATP to satisfy the immediate energy requirements for immune cell activation and proliferation; its metabolic intermediates provide precursors for synthesizing inflammatory mediators and reactive oxygen species, while accumulated lactate itself possesses signaling functions, collectively shaping and amplifying the pro-inflammatory microenvironment (43–46). On the other hand, for neurons with high energy dependence, the competitive glucose consumption by surrounding cells coupled with the inefficient energy output of glycolysis exacerbates cerebral energy crisis, whereas massive lactate accumulation induces local tissue acidosis, further impairing neuronal function (47–49).

The metabolic shift toward glycolysis fundamentally alters the downstream fate of pyruvate, directly impacting tricarboxylic acid (TCA) cycle dynamics [A/I]. Rather than entering mitochondria for complete oxidation, accumulating pyruvate is preferentially converted to lactate, simultaneously depriving the TCA cycle of its primary substrate and creating a metabolic bottleneck that perpetuates oxidative stress. This interconnected metabolic rewiring highlights that glycolytic enhancement and TCA cycle dysfunction are not isolated phenomena but represent coordinated adaptations to inflammatory stress.

#### 2.1.2 Decoupling of the tricarboxylic acid cycle and oxidative phosphorylation

Building upon these observations, further investigations reveal that decoupling of the tricarboxylic acid (TCA) cycle and oxidative phosphorylation constitutes the central link of mitochondrial dysfunction in SAE. The TCA cycle serves as the hub connecting glucose, lipid, and amino acid metabolism, with its reducing equivalents essential for driving the mitochondrial electron transport chain and oxidative phosphorylation (50, 51). In SAE, the TCA cycle undergoes “breakage” and functional remodeling. On one hand, TCA cycle intermediates are extensively withdrawn to support cellular anabolism and signal transduction—for instance, citrate is exported from mitochondria for fatty acid synthesis and epigenetic regulation, while succinate accumulation stabilizes HIF-1α, further amplifying the Warburg effect (52, 53). On the other hand, inflammatory mediators and oxidative stress directly compromise mitochondrial membrane integrity and inhibit electron transport chain complex activity, resulting in uncoupling of oxidative phosphorylation from electron transport. This not only precipitates sharp declines in ATP generation but also causes electron leakage and massive reactive oxygen species production, establishing a vicious cycle of “mitochondrial damage-oxidative stress” (54, 55). Metabolomics studies have confirmed significant disturbances in TCA cycle intermediates within brain tissue of SAE model animals (56).

TCA cycle dysfunction extends beyond energy metabolism, profoundly affecting lipid homeostasis [E]. When the cycle is interrupted, citrate accumulates and is exported to the cytoplasm via the citrate shuttle, becoming a substrate for *de novo* lipogenesis (57, 58). This metabolic rerouting, coupled with impaired β-oxidation due to mitochondrial dysfunction, creates a lipid accumulation state that further compromises cellular membrane integrity and signaling functions. These observations suggest that energy crisis and lipid dysregulation in SAE represent interconnected facets of mitochondrial pathology rather than independent processes (59, 60).

#### 2.1.3 Lipid metabolism dysregulation

Beyond disturbances in energy metabolic pathways, lipid metabolism dysregulation represents another critical characteristic of SAE, with impacts extending from cellular structural damage to signaling network imbalance (35, 60). The brain is the most lipid-rich organ; lipids not only constitute the fundamental scaffold of cell membranes but also extensively participate in complex signal transduction (34, 61). In SAE, lipid metabolism dysregulation manifests at multiple levels. First, membrane lipid degradation correlates with blood-brain barrier disruption. Phospholipase A2 and other hydrolases are activated to degrade phospholipids in neuronal and glial cell membranes, releasing arachidonic acid and other precursors that can be metabolized into potent pro-inflammatory mediators, exacerbating neuroinflammation and increasing blood-brain barrier permeability (35, 61). Second, fatty acid oxidation impairment occurs. Mitochondrial dysfunction obstructs β-oxidation of long-chain fatty acids, not only reducing energy sources but potentially leading to accumulation of lipotoxic substances such as acylcarnitines—one of the significantly altered metabolic pathways confirmed in plasma metabolomics studies of SAE patients (56, 62, 63). Furthermore, signaling lipid imbalance cannot be overlooked. Levels of lysophosphatidylcholine and other signaling lipid molecules undergo substantial changes; these molecules can influence neurotransmitter release, glial cell activation, and cell survival, with their imbalance disrupting the delicate signaling networks within the brain (64–66). Integrative transcriptomic and metabolomic studies have also identified significant lipid metabolic pathway dysregulation in the hippocampus of SAE mice (35).

Lipid metabolic disturbances are inextricably linked to amino acid metabolism through shared metabolic intermediates and compartmentalization [E] (67). Mitochondrial dysfunction, which impairs fatty acid oxidation, simultaneously compromises the catabolism of branched-chain amino acids that require the same organelle for their complete oxidation. Furthermore, altered membrane lipid composition affects the activity and localization of amino acid transporters, disrupting the delicate balance of neurotransmitter precursors. This metabolic convergence underscores the systemic nature of SAE-associated metabolic rewiring (68–70).

#### 2.1.4 Amino acid metabolism disturbance

Accompanying lipid metabolism dysregulation, amino acid metabolism disturbance directly disrupts the balance of neurotransmitter systems and the supply of metabolic fuels. Amino acids play multiple roles in the brain: serving as building blocks for protein synthesis, as neurotransmitters or their precursors, and as supplementary fuels for energy metabolism (71–73). Amino acid metabolism disturbance in SAE is both universal and complex. Most critically, imbalance between excitatory and inhibitory neurotransmitters manifests primarily as elevated glutamate levels, which may induce excitotoxicity and constitute an important mechanism of neuronal death (74, 75). Simultaneously, sepsis is frequently complicated by hepatic dysfunction; coupled with increased blood-brain barrier permeability, this enables substantial entry of aromatic amino acids from blood into the brain, where they compete with endogenous amino acids for transport carriers and may be metabolized into “false neurotransmitters” that interfere with normal synaptic transmission, resulting in consciousness disturbances (76–79). Additionally, consumption of branched-chain amino acids warrants attention: peripheral tissues such as muscle degrade proteins to release branched-chain amino acids as energy sources during sepsis, leading to decreased circulating levels of these amino acids, which may affect cerebral energy supply and neurotransmitter synthesis. Multiple metabolomics studies have identified amino acid metabolism among the most significantly affected pathways in SAE (80–83).

### 2.2 Specific metabolic reprogramming of three key brain cell types

The aforementioned global metabolic disturbances ultimately drive SAE pathogenesis by influencing the metabolic phenotypes of specific brain cells. Microglia, neurons, and astrocytes within the brain form a tightly integrated “neurovascular unit,” each undergoing unique metabolic reprogramming and interacting to collectively determine disease trajectory (4, 19, 35, 84). The metabolic reprogramming of key brain cell types is summarized in Table 1.

**Table 1:** Specific metabolic reprogramming of key brain cell types in SAE.

Cell type	Metabolic characteristics	Pathological significance	References
Microglia	Enhanced glycolysis (Warburg effect) driven by mTOR/HIF-1α pathway.	Polarization toward a pro-inflammatory phenotype, releasing IL-1β and mediating NLRP3 pyroptosis.	(19, 82–85, 87)
Neurons	Highly dependent on mitochondrial oxidative phosphorylation; limited metabolic flexibility.	Energy crisis, ion pump failure, synaptic dysfunction, and cell death.	(88–92, 101–106)
Astrocytes	Enhanced glycolysis; potential lactate shuttle to neurons; can transition to a neurotoxic A1 phenotype.	Impaired glutamate uptake exacerbates excitotoxicity; contributes to BBB disruption.	(107–111, 113, 118)

#### 2.2.1 Microglia: metabolic coupling of immune activation

As resident immune cells of the central nervous system, microglia play a central role in SAE neuroinflammation, with their activation states tightly coupled to metabolic phenotypes (85–87). Upon stimulation by pathogen-associated molecular patterns such as lipopolysaccharide, microglia rapidly switch from the oxidative phosphorylation-dependent metabolic mode characteristic of the resting state to a pro-inflammatory phenotype dominated by glycolysis. This process is driven by the mTOR/HIF-1α signaling axis; enhanced glycolysis provides necessary bioenergy and metabolic intermediates for synthesizing pro-inflammatory factors and generating reactive oxygen species (88–91). In contrast, tissue-reparative anti-inflammatory phenotypes depend on intact TCA cycle and fatty acid oxidation. Recent studies have additionally revealed that pyroptosis, an inflammatory form of programmed cell death, is activated in SAE microglia; pharmacological inhibition of pyruvate dehydrogenase kinase 4 can reverse the Warburg effect in microglia, thereby suppressing NLRP3 (NOD-, LRR- and pyrin domain-containing protein 3); SOFA (Sequential Organ Failure Assessment) inflammasome-mediated pyroptosis, reducing neuronal death, and improving cognitive function (92, 93). This provides direct evidence for targeting microglial metabolism to control neuroinflammation, demonstrating that modulating microglial metabolic states to shift from pro-inflammatory glycolytic phenotypes toward anti-inflammatory oxidative phosphorylation phenotypes represents a highly attractive therapeutic strategy.

#### 2.2.2 Neurons: energy crisis in highly differentiated terminal cells

**Figure 1:**
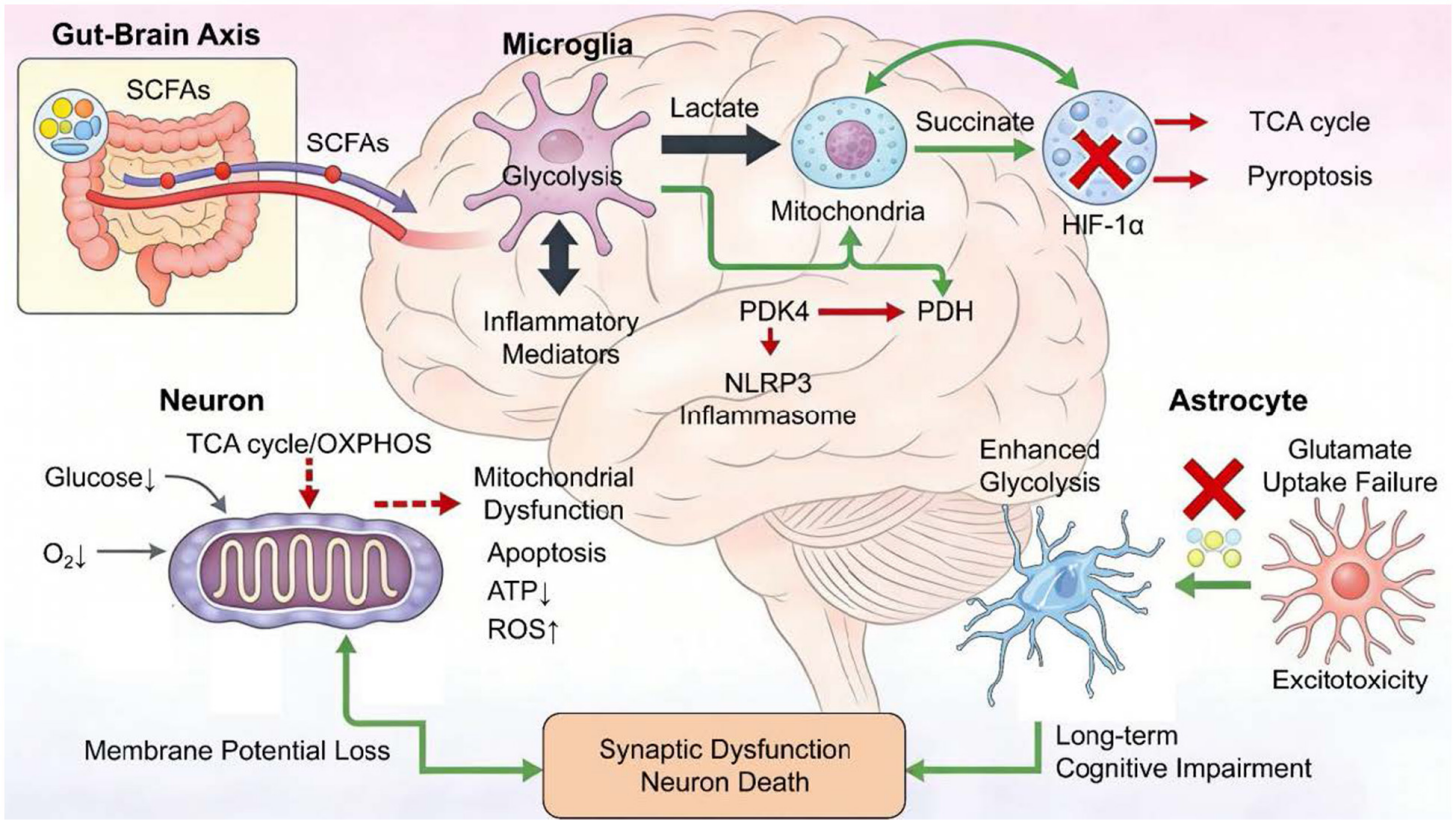
Metabolic reprogramming drives cell-type-specific pathogenic mechanisms in sepsis-associated encephalopathy. The schematic illustrates the cell-type-specific metabolic alterations driving sepsis-associated encephalopathy (SAE) pathogenesis [established mechanisms], integrating the gut-brain axis and downstream cognitive outcomes, wherein peripheral infection and gut dysbiosis-derived metabolites signal via the gut-brain axis [established mechanisms] to activate microglia, in which the mTOR/HIF-1α pathway drives a metabolic shift toward aerobic glycolysis (Warburg effect) [established mechanisms] characterized by enhanced glucose uptake and lactate production [established mechanisms], fueling NLRP3 inflammasome activation [established mechanisms] and leading to IL-1β release [established mechanisms] and PDK4-mediated pyroptosis [proposed mechanism] that perpetuates neuroinflammation, while neurons simultaneously face reduced delivery of glucose and oxygen [established mechanisms] coupled with intrinsic mitochondrial dysfunction [established mechanisms] that disrupts the TCA cycle [established mechanisms] and uncouples oxidative phosphorylation [established mechanisms], causing ATP depletion [established mechanisms] and reactive oxygen species overproduction [established mechanisms] that impair ion pump activity [established mechanisms] and trigger apoptosis [established mechanisms], and astrocytes concurrently exhibit impaired ATP-dependent glutamate uptake [established mechanisms] leading to synaptic glutamate accumulation and excitotoxic neuronal injury [established mechanisms] while potentially transitioning to a neurotoxic A1 phenotype [emerging concept] that further compromises blood-brain barrier integrity [established mechanisms], with the synergistic effects of microglial neuroinflammation, neuronal energy failure, and astrocyte dysfunction ultimately culminating in synaptic impairment and neuronal death [established mechanisms] that manifests as long-term cognitive dysfunction in SAE survivors [established mechanisms], with abbreviations including HIF-1α, hypoxia-inducible factor-1α; IL-1β, interleukin-1β; mTOR, mammalian target of rapamycin; NLRP3, NOD-, LRR- and pyrin domain-containing protein 3; OxPhos, oxidative phosphorylation; PDK4, pyruvate dehydrogenase kinase 4; PDH, Pyruvate Dehydrogenase Complex; ROS, reactive oxygen species; and TCA, tricarboxylic acid.

Unlike the plasticity of microglia, neurons—as highly differentiated terminal cells—require enormous energy consumption for electrical activity and maintenance of ion gradients, rendering them extremely dependent on efficient mitochondrial oxidative phosphorylation (94–98). In SAE, neurons face dual assaults from internal and external environments, plunging into profound energy crisis. Regarding external supply, systemic hypotension, hypoxemia, and cerebral microcirculatory disturbances directly reduce delivery of the two most critical energy substrates: glucose and oxygen (99–103). Regarding internal production, mitochondria become dysfunctional under attack by oxidative stress and inflammatory mediators, drastically diminishing ATP synthetic capacity (104–106). Compared with glial cells, neurons possess limited capacity to utilize alternative energy sources such as ketone bodies or fatty acids, and their glycolytic capacity is insufficient to compensate for deficits in oxidative phosphorylation. This severe energy deficit directly precipitates neuronal dysfunction and death: inability to maintain membrane potential leads to ion pump failure and cellular edema; synaptic transmission interruption causes abnormal signal processing; calcium overload activates multiple degradative enzymes, ultimately triggering apoptotic or necrotic programs (107–112). Therefore, protecting neuronal mitochondrial function and restoring energy supply constitutes the core of neuroprotective strategies in SAE. Metabolic reprogramming of key brain cell types is illustrated in Figure 1.

#### 2.2.3 Astrocytes: functional reprogramming from “guardians” to “destroyers”

Within the brain's cellular network, astrocytes—as the most abundant glial cell type—play multiple critical “housekeeping” roles, from maintaining blood-brain barrier integrity and providing nutritional support to neurons, to buffering ion and neurotransmitter concentrations (113–115). In SAE, astrocytes similarly undergo complex metabolic and functional reprogramming, with their roles potentially shifting from “guardians” to “destroyers.” Under inflammatory stimulation, astrocytes become activated and undergo reactive proliferation; their metabolic mode may also enhance glycolysis, providing energy for self-proliferation while potentially supplying energy substrates to damaged neurons through “astrocyte-neuron lactate shuttle,” reflecting their potential protective effects (116–118). However, analogous to microglial polarization, activated astrocytes can be classified into neurotoxic A1 phenotypes and neuroprotective A2 phenotypes, with A1 astrocytes releasing multiple substances toxic to neurons and oligodendrocytes (119, 120). Although current research on their metabolic characteristics remains insufficient, this functional differentiation likely undergoes regulation by specific metabolic pathways (121–123). Particularly importantly, astrocytes are responsible for uptake of excess glutamate from the synaptic cleft to prevent excitotoxicity; this ATP-dependent process becomes severely impaired during SAE energy crisis, leading to glutamate accumulation in the synaptic cleft and thereby exacerbating neuronal damage (116, 124, 125). Despite the need for further in-depth research on specific metabolic reprogramming of astrocytes in SAE, their role as core components of the neurovascular unit ensures that alterations in their metabolic states will inevitably exert profound influences on SAE disease course (116, 117).

#### 2.2.4 Peripheral immune cell infiltration and brain metabolic microenvironment remodeling

Importantly, recent metabolomic studies have identified succinate as a key metabolite linking peripheral inflammation to brain dysfunction in sepsis [A] (126). Elevated circulating succinate levels correlate with SAE severity and may serve as a metabolic signal coordinating immune cell function across the peripheral-central interface. These findings highlight that SAE metabolic disturbances are not confined to the CNS but represent systemic metabolic dysregulation manifesting in the brain (35, 126).

T lymphocytes, upon antigen-independent activation during sepsis, demonstrate metabolic reprogramming toward glycolysis to support rapid proliferation and cytokine production [A]. The presence of metabolically active T cells in perivascular spaces may influence astrocytic and microglial metabolic states through paracrine signaling (28, 127).

Neutrophils, characterized by their reliance on aerobic glycolysis for effector functions including NETosis, release extracellular traps enriched in oxidizing enzymes and proteases that further perturb local metabolic homeostasis [A]. Their brief but intense metabolic activity creates localized regions of hypoxia and acidification that stress neighboring neural cells (128, 129).

Circulating monocytes recruited to the brain undergo rapid metabolic adaptation upon infiltration. Recent single-cell analyses reveal that infiltrating monocyte-derived macrophages exhibit enhanced glycolytic activity compared to their circulating counterparts, competing with resident microglia for glucose substrates while amplifying local inflammatory signals [A] (130). This metabolic competition may exacerbate neuronal energy deprivation during critical disease phases.

While the preceding discussion focuses on intrinsic brain cells, accumulating evidence indicates that infiltrating peripheral immune cells significantly contribute to and reshape the brain's metabolic microenvironment during SAE [A/H] (130). Under systemic inflammatory conditions, monocytes, neutrophils, and T cells traverse the compromised blood-brain barrier, bringing their distinct metabolic profiles into the CNS parenchyma (131, 132).

### 2.3 Gut-brain axis metabolic dialogue: “adding fuel to the fire” from distal organs

Building upon in-depth analysis of intracerebral cellular metabolic reprogramming, recent research has revealed that metabolic disturbances in SAE do not occur in isolation within the cranium but are profoundly influenced by metabolic states of distal organs, particularly the intestine, with the gut-brain axis playing a role of “adding fuel to the fire” (133, 134). Sepsis frequently causes impaired intestinal barrier function, or “leaky gut,” accompanied by dysbiosis, producing far-reaching systemic effects that propagate to the brain through metabolic pathways. Metabolites produced by intestinal microbiota, such as short-chain fatty acids, secondary bile acids, and indole derivatives, possess important immunomodulatory and neuromodulatory functions (135–137). Sepsis-induced microbiota disturbance alters the profile of these metabolites—for instance, reduced production of anti-inflammatory and neuroprotective short-chain fatty acids, with potential increases in harmful metabolites. These altered metabolites can reach the brain through the circulatory system, directly or indirectly influencing brain cell function. They can modulate peripheral immune cell activity, affecting their migration to the brain, and can cross the compromised blood-brain barrier to directly act upon microglia and astrocytes, regulating their activation states and metabolic phenotypes (133, 138–140). A study in a sepsis mouse model explicitly revealed functional disturbance of the “gut microbiota-hippocampus-metabolite axis,” confirming the association between intestinal dysbiosis and hippocampal dysfunction and identifying related metabolite alterations (141). This suggests that when considering intervention strategies for SAE, metabolic dialogue along the gut-brain axis must be incorporated into consideration—for example, indirectly intervening in brain function through modulation of intestinal microbiota or supplementation of key microbial metabolites—opening new possibilities for future SAE prevention and treatment (133, 142).

## 3 Biomarker exploration driven by metabolic reprogramming

The clinical diagnosis of SAE currently remains predominantly dependent on clinical manifestations and exclusionary strategies, with a notable absence of molecular biomarkers possessing both high sensitivity and strong specificity. This limitation not only delays the implementation of early intervention but also restricts precise subject selection in clinical trials and objective assessment of therapeutic efficacy. As the terminal output products of cellular functional states and pathophysiological processes, metabolites inherently possess ideal attributes for serving as disease markers (56, 143). Therefore, systematically mining characteristic metabolites capable of reflecting core pathological alterations in SAE from the perspective of metabolic reprogramming opens novel research avenues for early identification and mechanistic stratification of this disease. Key metabolomics-driven biomarkers for SAE are summarized in Table 2.

**Table 2:** Metabolomics-driven biomarkers for SAE.

Metabolic pathway	Representative metabolites	Pathological significance	References|evidence level
Fatty acid oxidation	Acylcarnitines	Mitochondrial dysfunction; impaired fatty acid oxidation.	(57, 61, 147)[A/H]
Phospholipid metabolism	Lysophosphatidylcholines (LysoPCs)	Membrane structural damage; signaling imbalance.	(62–64, 147)[A/H]
TCA cycle	Succinate	Stabilizes HIF-1α, promoting a pro-inflammatory state.	(85, 149, 150)[A/I]
Aromatic amino acid metabolism	4-hydroxyphenylacetic acid	Potential marker for gut dysbiosis and neurotransmitter synthesis abnormalities.	(151, 152)[H]
One-carbon metabolism	Betaine, folate metabolites	Disturbances in methylation processes, affecting neuronal function.	(147)[A]

### 3.1 Limitations of traditional biomarkers

Retrospective examination of prior research reveals that traditional candidate biomarkers have primarily concentrated on neuronal injury markers such as neuron-specific enolase, glial activation or injury markers such as S100B protein and glial fibrillary acidic protein, and various inflammatory cytokines such as interleukin-6 (144–146). However, these markers universally face significant limitations in clinical application and mechanistic interpretation. First, the issue of non-specificity is particularly prominent; the aforementioned markers can be elevated in multiple central nervous system injuries including traumatic brain injury and stroke, making precise targeting of SAE difficult (147–149). Second, the release of these markers typically occurs following structural neuronal injury or glial activation, and their elevated levels may miss the optimal time window for early intervention (150–152). Furthermore, the *in vivo* clearance of protein-based markers is highly dependent on renal function, whereas sepsis patients frequently develop acute kidney injury, which directly interferes with clinical interpretation of plasma marker concentrations (153–156). Most critically, traditional markers largely reflect the final outcomes of injury rather than revealing the dynamic pathological mechanisms driving this injury process, thus providing limited guidance for mechanism-directed intervention strategies (157, 158).

#### 3.1.1 Critical barriers to clinical translation of metabolic biomarkers

**Linking biomarkers to therapeutic decisions:** For metabolic biomarkers to achieve clinical impact, they must inform therapeutic decisions beyond mere diagnosis. Future research should focus on identifying metabolite signatures that predict response to specific metabolic interventions (e.g., glycolytic inhibitors, mitochondrial protectors), enabling biomarker-guided personalized therapy rather than one-size-fits-all approaches.

**Therapeutic and nutritional interference:** Standard ICU interventions—including vasoactive medications, sedatives, continuous renal replacement therapy, and parenteral nutrition—substantially alter metabolite profiles. Without standardized sampling protocols accounting for these confounders, inter-study comparability and clinical utility remain limited.

**Specificity for SAE vs. sepsis severity:** A fundamental challenge lies in distinguishing metabolite alterations specific to brain dysfunction from those reflecting systemic sepsis severity. Many candidate biomarkers identified in SAE studies correlate equally well with SOFA (Sequential Organ Failure Assessment); scores or lactate levels, raising questions about their added diagnostic value beyond general severity assessment (159, 160).

**Confounding by organ dysfunction:** Sepsis commonly involves multi-organ dysfunction, particularly acute kidney injury, which profoundly affects metabolite clearance and plasma concentrations [H]. Elevated acylcarnitines or amino acid derivatives may reflect renal excretory impairment rather than specific brain metabolic pathology, complicating clinical interpretation.

**Temporal dynamics and sampling windows:** Metabolite profiles exhibit rapid temporal evolution during sepsis progression. The optimal timing for sample collection—whether at ICU admission, at peak illness severity, or during resolution phases—remains undefined. Single time-point measurements may miss critical metabolic transitions or capture transient fluctuations rather than sustained pathological signatures.

**Point-of-care applicability:** Current metabolomics platforms typically require sophisticated mass spectrometry or NMR (nuclear magnetic resonance); AMP (adenosine monophosphate) facilities with specialized personnel, limiting their deployment in emergency department settings where early SAE detection would be most valuable. Development of rapid, bedside-compatible assays for priority metabolites remains an unmet need.

### 3.2 Metabolomics-driven discovery of differential metabolites

With the rapid advancement of high-throughput, high-sensitivity metabolomics detection technologies—particularly the widespread application of mass spectrometry and nuclear magnetic resonance techniques—researchers can now simultaneously detect hundreds to thousands of small-molecule metabolites in blood, cerebrospinal fluid, urine, and even tissue samples, thereby capturing systemic metabolic disturbance characteristics during SAE occurrence and progression through a panoramic perspective (56, 161, 162). In recent years, multiple cutting-edge metabolomics-based studies have identified a series of differential metabolites with potential biomarker value and their associated pathways in SAE patients and animal models, providing novel data support for understanding the pathological essence of SAE (56, 163).

Notably, a prospective study conducted plasma untargeted metabolomics analysis in SAE patients, identifying multiple significantly perturbed metabolic pathways including acylcarnitine metabolism, lysophosphatidylcholine metabolism, betaine metabolism, folate biosynthesis, and cytochrome P450 drug metabolism pathways. The study further screened sixty-four potential differential metabolites, providing a rich candidate molecular library for constructing SAE diagnostic or prognostic prediction models (163). The alterations in these metabolites, respectively point to core pathological processes such as mitochondrial fatty acid oxidation impairment, cell membrane lipid degradation and signaling imbalance, and one-carbon unit metabolism disturbance, highly consistent with the aforementioned mechanistic descriptions of metabolic reprogramming. Additional independent studies have reported that significantly enriched differential metabolic pathways in SAE involve iminoglycine metabolism, aspartate and alanine metabolism, pantothenate and glutamate metabolism, coenzyme A biosynthesis, and linoleic acid, betaine, and hypoxanthine metabolism, once again confirming the central positions of amino acid metabolism disturbance, energy metabolism impairment, and lipid metabolism imbalance in SAE pathogenesis (35, 56).

Particularly important is that certain metabolites are themselves embedded within specific pathophysiological pathways, with their level changes capable of directly reflecting mechanistic disease progression (164). For instance, succinate, as a key molecule connecting the tricarboxylic acid cycle with inflammatory signal transduction, its elevated levels in SAE may predict activation of mitochondrial stress states and hypoxia-inducible factor-1α-mediated inflammatory response cascades (91, 165, 166). Additionally, 4-hydroxyphenylacetic acid has been identified as a promising biomarker, with its plasma levels demonstrating significant correlation with the severity of consciousness disturbance in SAE patients, potentially reflecting abnormalities in aromatic amino acid metabolism against the background of intestinal microbiota disturbance. These findings suggest that metabolomics-driven biomarker exploration can provide not only novel diagnostic tools but, more importantly, the identified metabolites themselves carry mechanistic information regarding disease states, capable of revealing SAE heterogeneity characteristics at the molecular level—an advantage unattainable by traditional markers (167, 168). Therefore, conducting in-depth SAE biomarker research with metabolic reprogramming as the theoretical framework combined with metabolomics technology holds promise for providing critical molecular evidence for achieving early diagnosis, precise stratification, and individualized treatment of this disease.

### 3.3 Multi-omics integration: the inevitable path toward precision medicine

Although single-omics technologies can reveal pathological features of SAE at specific molecular levels, they capture only one facet of complex biological processes. To achieve more comprehensive and systematic resolution of the molecular essence of SAE and to genuinely advance biomarker discovery toward clinical translation, multi-omics integration analysis has become an inevitable trend in this research field (169, 170). Through systematic integration of genomic, transcriptomic, proteomic, and metabolomic data, researchers can construct complete molecular regulatory networks spanning from upstream gene regulation, through intermediate functional protein expression, to downstream metabolite output, thereby identifying biomarker combinations and potential therapeutic targets with enhanced robustness and causal relevance (171–173).

In recent years, the application of multi-omics integration strategies in SAE research has achieved significant progress. Taking the joint analysis of transcriptomics and metabolomics as an example, a study successfully mapped the molecular landscape of the disease through parallel omics detection of hippocampal tissue from SAE mice. This study identified eighty-one differentially expressed metabolites and over one thousand seven hundred differentially expressed genes, subsequently revealing intrinsic associations among neuroinflammatory activation, downregulation of multiple core metabolic pathways, and synaptic functional impairment. Such integrative analysis not only validated the systemic dysregulation of lipid, amino acid, glucose, and nucleotide metabolism in SAE but also traced upstream transcriptional regulatory events driving these metabolic alterations, providing a deeper causal perspective for understanding disease pathogenesis (174).

The joint analysis of proteomics and metabolomics offers a more direct chain of evidence for mechanistic validation of metabolic regulation. One study integrated plasma proteomic and metabolomic data from sepsis patients, identifying amino acid metabolism disturbance as a core pathological characteristic throughout the SAE disease course, and specifically highlighting the potential role of aberrant alterations in the pentose phosphate pathway in disease occurrence and progression. Proteomics can precisely identify expression-level changes in key enzymes regulating metabolic pathways, while metabolomics validates the functional consequences resulting from these changes; their complementary combination substantially enhances the reliability of research conclusions and mechanistic explanatory power (175, 176).

Future-oriented research paradigms are increasingly incorporating machine learning and artificial intelligence algorithms into the processing and interpretation of multi-omics data. By integrating clinical phenotypic data with multi-dimensional omics information, researchers aim to construct complex predictive models capable of early prediction of SAE risk, precise assessment of disease severity, and even prediction of long-term cognitive prognosis. This data-driven integrative analytical strategy not only facilitates precise stratification of SAE patients but also provides scientific foundations for the formulation of personalized treatment protocols (8, 116, 177–179).

In summary, biomarker research driven by the perspective of metabolic reprogramming is undergoing a profound transformation from discovery of individual differential metabolites toward construction of biomarker combinations and predictive models guided by multi-omics integration. This evolution in research paradigms not only holds promise for reshaping clinical diagnostic and therapeutic pathways for SAE but also lays a solid scientific foundation for the ultimate realization of precision medicine in this field.

## 4 Therapeutic strategies targeting metabolic reprogramming

Given the central driving role of metabolic reprogramming in the pathological progression of SAE, direct intervention in specific metabolic pathways and correction of systemic metabolic disturbances have emerged as highly promising new therapeutic directions in this field. This strategy not only requires us to re-examine and optimize existing clinical supportive treatments from a novel metabolic perspective but also encompasses the development and application of a series of new modulators targeting metabolic nodes (19, 27, 169). Specific therapeutic strategies are summarized in Table 3.

**Table 3:** Therapeutic strategies targeting metabolic reprogramming in SAE.

Intervention type	Representative agent/strategy	Mechanism of action	References|evidence level|major limitations
Metabolic modulator	Metformin	Activates AMPK, inhibits mTOR/HIF-1α axis, reducing neuroinflammation.	(176–184)[A]|lactic acidosis risk; BBB penetration unclear
Antioxidant and mitochondrial protector	Melatonin	Activates SIRT1/NRF2, inhibits NLRP3, protects mitochondrial function.	(187–193)[A] vs. [H-]|ICU trials negative; inconsistent PK
Selective antioxidant	Hydrogen gas	Scavenges hydroxyl radicals, stabilizes BBB, modulates mitochondrial function.	(196–204)[A]|no human SAE data; delivery standardization needed
Metabolic redirector	Dichloroacetate (DCA)	Inhibits PDK4, activates PDH, reverses the Warburg effect, inhibits pyroptosis.	(87, 207, 208)[A]|neurotoxicity concerns; limited efficacy data
Supportive care	Precision nutrition	Dynamically adjusts carbohydrate, lipid, and amino acid ratios to alleviate metabolic burden.	(166, 169, 170)[E]|optimal composition undefined; delivery challenges

### 4.1 Re-evaluating standard supportive care from a metabolic perspective

Reflecting on metabolic reprogramming, many standardized supportive treatments for septic patients in intensive care units inherently possess profound metabolic intervention significance. Taking glycemic control as an example, sepsis frequently induces stress hyperglycemia, which not only exacerbates tissue damage through oxidative stress pathways but more importantly provides excessive glycolytic substrates for the Warburg effect in immune cells, thereby “fueling” the inflammatory response (42, 180–183). Therefore, strict glycemic management exerts anti-inflammatory effects largely by restricting fuel supply for pro-inflammatory metabolism (184). Nutritional support similarly faces complex metabolic trade-offs: overfeeding may aggravate the already overwhelmed mitochondrial burden, whereas undernutrition intensifies energy crisis and muscle catabolism (185, 186). Future precision nutrition strategies require dynamic adjustment of carbohydrate, lipid, and specific amino acid proportions based on patients' metabolic phenotypes, aiming to provide appropriate energy substrates for the damaged brain while avoiding exacerbation of pathological metabolic reprogramming. Furthermore, aggressive fluid resuscitation and vasoactive agent administration fundamentally target restoration of macroscopic and microscopic tissue perfusion, ensuring effective delivery of critical metabolic substrates such as oxygen and glucose, thereby alleviating cellular energy crisis at its source (187–189). Thus, integrating the theoretical framework of metabolic reprogramming into conventional treatments deepens understanding of their intrinsic mechanisms and provides theoretical guidance for achieving personalized management based on individual metabolic characteristics.

### 4.2 Novel metabolic modulators in preclinical models

Building upon in-depth understanding of existing treatments, a series of drugs capable of precisely regulating cellular metabolism have demonstrated encouraging therapeutic potential in preclinical animal models of SAE in recent years (39, 190, 191).

#### 4.2.1 Metformin

Metformin, as a first-line therapeutic agent for type 2 diabetes mellitus, has activation of adenosine monophosphate-activated protein kinase (AMPK) as one of its core mechanistic actions. AMPK serves as the cellular “energy sensor,” activated when ATP levels decline, restoring energy homeostasis through inhibiting anabolism and promoting catabolism (192–195). In the SAE context, AMPK activation effectively suppresses glycolysis driven by the mTOR/HIF-1α axis, thereby attenuating pro-inflammatory responses in microglia (196, 197). Numerous animal experiments demonstrate that metformin can alleviate sepsis-induced neuroinflammation, neuronal apoptosis, and blood-brain barrier injury while significantly improving cognitive function; its protective effects also involve multiple mechanisms including antioxidant activity and epigenetic modification (198–200). Although some retrospective clinical studies suggest metformin may improve prognosis in septic patients, its use during the acute critical phase requires vigilance regarding lactic acidosis risk, and confirmation from large-scale prospective clinical trials remains lacking (201, 202).

**Clinical translation status:** Currently not recommended for acute SAE treatment outside clinical trials.

**Major translational limitations:** (1) The risk of lactic acidosis in critically ill patients with hemodynamic instability or renal dysfunction contraindicates metformin use during acute sepsis; (2) BBB penetration of metformin under inflammatory conditions is incompletely characterized; (3) Optimal dosing and timing relative to sepsis onset remain undefined.

**Evidence assessment:** Current support for metformin in SAE derives predominantly from animal models [A], with limited clinical data specifically examining neurological outcomes in septic patients. A retrospective cohort study suggested improved mortality in diabetic septic patients receiving metformin, though neurocognitive endpoints were not assessed [H].

#### 4.2.2 Melatonin

Melatonin, as a potent endogenous antioxidant, exhibits diverse neuroprotective mechanisms. From a metabolic perspective, melatonin not only directly scavenges free radicals and activates antioxidant pathways such as SIRT1/NRF2 (sirtuin 1/nuclear factor erythroid 2-related factor 2); but also attenuates inflammatory responses through NLRP3 inflammasome inhibition; more critically, it protects mitochondrial function and reduces energy failure resulting from oxidative stress (203–206). In various SAE animal models, melatonin has been proven effective in reducing cerebral edema, cellular apoptosis, and cognitive impairment (207–209). Given its extremely high safety profile, small-scale clinical trials have already demonstrated its potential in treating multi-organ injury in sepsis, though optimal dosing and administration timing for SAE treatment await determination through large-scale clinical studies (210, 211).

**Clinical translation status:** Evidence insufficient to support routine clinical use; may warrant further investigation in specifically selected SAE subpopulations.

**Major translational limitations:** (1) Inconsistent pharmacokinetics in critically ill patients; (2) Optimal dosing (high-dose vs. physiological replacement) remains debated; (3) Timing of administration relative to injury onset may be critical but is undefined; (4) Conflicting clinical trial results question the generalizability of animal findings to human ICU populations.

**Evidence assessment:** Robust neuroprotective effects are demonstrated in multiple SAE animal models [A]. However, recent randomized controlled trials in critically ill patients have yielded disappointing results. The MENDS2 (Maximizing the Efficacy of Sedation and Reducing Neurological Dysfunction and Mortality in Septic Patients with Acute Respiratory Failure) trial and other studies examining melatonin or melatonin receptor agonists for delirium prevention in ICU settings showed no significant benefit in reducing acute neurological dysfunction [H]. These negative findings highlight the translational gap between preclinical promise and clinical efficacy.

#### 4.2.3 Hydrogen gas

Hydrogen gas, as a selective antioxidant, possesses the unique advantage of effectively neutralizing the most toxic hydroxyl radicals and peroxynitrite anions while minimally affecting reactive oxygen species with normal physiological signaling functions (212). Its protective mechanisms additionally include activation of the Nrf2 antioxidant pathway, NLRP3 inflammasome inhibition, blood-brain barrier stabilization, and mitochondrial function modulation (213–218). Animal experiments consistently demonstrate that hydrogen inhalation or consumption of hydrogen-rich water significantly improves survival rates and neurological outcomes in septic animals (219, 220). The biological safety of hydrogen has been confirmed in clinical trials for other diseases; as a simple and inexpensive therapeutic modality, its clinical translation potential in SAE is substantial, though targeted clinical research data remain currently unavailable (221, 222).

**Clinical translation status:** Preclinical stage; clinical trials in SAE or general sepsis populations needed before therapeutic consideration.

**Major translational limitations:** (1) Delivery methods (inhalation vs. hydrogen-rich water) require standardization; (2) Optimal concentration, duration, and timing of administration undefined; (3) Safety profile in critically ill, ventilated patients requires formal evaluation; (4) Regulatory pathways for hydrogen as a therapeutic agent remain unclear in most jurisdictions.

**Evidence assessment:** All current evidence for hydrogen therapy in SAE derives from animal models [A]; no human SAE-specific studies have been published. Promising findings in rodent CLP and LPS models demonstrate improved survival and reduced brain injury biomarkers.

#### 4.2.4 Dichloroacetate

The mechanism of dichloroacetate (DCA) exemplifies precise linkage from metabolic correction to cellular protection. DCA is an inhibitor of pyruvate dehydrogenase kinase (PDK), capable of activating pyruvate dehydrogenase (PDH)—the key rate-limiting enzyme converting the glycolytic product pyruvate into acetyl-CoA for entry into the tricarboxylic acid cycle—through PDK inhibition. Consequently, DCA forcibly redirects cellular metabolism from glycolysis toward oxidative phosphorylation (223, 224). A pioneering study discovered that DCA specifically inhibits pyroptosis in microglia in SAE models, with the mechanism precisely involving reversal of the Warburg effect through PDK4 isoform inhibition, thereby blocking NLRP3 inflammasome activation. This research precisely linked metabolic modulation to specific cell death programs, providing robust theoretical justification for DCA application (93). However, DCA was previously used for treating inherited lactic acidosis, and its neurotoxicity with long-term administration represents the primary obstacle to clinical application; nevertheless, short-term use in acute diseases such as SAE may offer a more favorable risk-benefit ratio, though rigorous clinical trials remain necessary to evaluate its safety and efficacy (225, 226).

**Clinical translation status:** Early preclinical; significant safety and efficacy data required before clinical consideration.

**Major translational limitations:** (1) Historical concerns regarding peripheral neuropathy with chronic DCA administration, though short-term use in acute illness may present acceptable risk-benefit profile; (2) BBB penetration characteristics in sepsis-related BBB disruption unknown; (3) Potential for lactic acidosis in patients with thiamine deficiency; (4) No pharmaceutical-grade formulation readily available for clinical use.

**Evidence assessment:** The evidence base for DCA in SAE consists of a single mechanistic study demonstrating microglial pyroptosis inhibition through PDK4 blockade [A]. No broader efficacy studies or dose-response analyses in SAE models have been published.

### 4.3 Challenges and future directions in clinical translation

Despite abundant achievements in preclinical research, successful translation of metabolic modulators into clinical therapies for SAE faces severe challenges. First is the issue of patient heterogeneity: the etiology, severity, and pathophysiological processes of sepsis are highly heterogeneous, and uniform intervention protocols may prove ineffective or even harmful for certain patients; future approaches must employ precise stratification based on biomarkers (227–231). Second is the determination of therapeutic time windows: metabolic reprogramming is a dynamic process—early enhancement of glycolysis may facilitate pathogen clearance, whereas sustained Warburg effects in later stages are deleterious; identifying optimal intervention timing is therefore critical (232, 233). Additionally, target specificity and off-target effects present substantial difficulties: many metabolic enzymes play divergent roles in different cell types, and systemic administration may produce unintended effects; developing drug delivery systems targeting specific cells represents an important future direction (234–236). Simultaneously, drugs targeting the central nervous system must effectively penetrate the blood-brain barrier, and the physicochemical properties of many candidate drugs limit their cerebral bioavailability (237–242). Finally, preclinical studies predominantly employ young, healthy inbred animals, which differ substantially from the complex, multi-comorbid ICU patients encountered clinically—this disparity constitutes a primary reason for drug failures in clinical trials (243–245). Future drug development should focus on developing novel metabolic modulators with higher cell-type and pathway specificity, utilizing advanced delivery systems such as nanotechnology to achieve targeted therapy, and conducting adaptive clinical trials based on precision biomarker stratification to bridge the translational gap from basic research to clinical application.

## 5 Research technologies and future perspectives

The continuous advancement of metabolic reprogramming research in SAE relies indispensably on the refined application of advanced research tools and prospective strategic planning. From optimized selection of animal models to breakthroughs in single-cell resolution multi-omics technologies, and further to systematic design of translational medicine pathways, each determinant critically influences the efficiency and feasibility of translating basic research discoveries into clinical practice (246–248). These technologies are summarized in Table 4.

**Table 4:** Research technologies and future perspectives in SAE.

Technology/direction	Applications and significance	Challenges and future outlook	References
Animal models	CLP model for polymicrobial sepsis; LPS model for mechanistic screening.	High inter-model variability; metabolic profiles influenced by anesthesia, strain, and circadian rhythms; requires multi-model validation.	(233–254)
Multi-omics integration	Constructs regulatory networks by integrating genomics, transcriptomics, proteomics, and metabolomics.	Data standardization and algorithmic complexity; requires interdisciplinary collaboration.	(255–266)
Single-cell metabolomics	Resolves cellular heterogeneity and reveals metabolic interactions within the microenvironment.	Technical bottlenecks: low metabolite content, need for high sensitivity, and loss of spatial information.	(267–284)
Translational pathways	Biomarker validation; drug repurposing; novel drug development; combination therapies.	Requires large-scale validation, precise patient stratification, and targeted drug delivery systems.	(285–298)

### 5.1 Advantages and limitations of commonly used animal models

The advantages and limitations of commonly used animal models constitute the cornerstone of metabolic research. The cecal ligation and puncture (CLP) model is widely recognized as the “gold standard” for sepsis research (249–251). Through surgical intestinal perforation, this model induces intraperitoneal polymicrobial infection and peritonitis, effectively simulating the pathophysiological processes of human sepsis, including hyperdynamic circulatory states, dynamic evolution of inflammatory responses, and multiple organ dysfunction. The model demonstrates relatively high reproducibility; researchers can precisely control infection severity by adjusting ligation length, needle gauge, and puncture frequency (251). However, the technical complexity of CLP procedures demands high surgical expertise, and variability in standardization across different laboratories may introduce result heterogeneity, while surgical trauma itself introduces additional inflammatory variables (252–255). In contrast, the lipopolysaccharide (LPS) model, administered via intraperitoneal or intravenous injection of the principal component of Gram-negative bacterial outer membranes, rapidly induces intense, predictable systemic inflammatory responses with extremely simple operation, controllable dosage, and excellent repeatability, rendering it suitable for high-throughput drug screening and preliminary mechanistic exploration (256–258). Nevertheless, the primary limitation of the LPS model lies in its simulation of pure, single-pathogen-molecule-driven “sterile inflammation,” lacking authentic infectious foci and pathogen proliferation processes; its inflammatory kinetics diverge substantially from clinical sepsis, and high-dose administration may even induce hypodynamic shock states inconsistent with clinical presentations (259–263). When conducting metabolic research, regardless of the model employed, investigators must exercise extreme caution in controlling the significant influences of anesthetics, surgical stress, animal strain, age, sex, and circadian rhythms on metabolic profiles, while recognizing the limited translational rate of animal model data to clinical applications; critical findings require rigorous validation in human specimens (264–269). The optimal strategy involves combining multiple models, utilizing respective advantages for cross-validation of key conclusions to enhance research reliability (270).

### 5.2 Necessity and challenges of multi-omics integration analysis

The necessity and challenges of multi-omics integration analysis are becoming increasingly prominent. Single-omics technologies are now insufficient to reveal the complex pathological processes of SAE; future research must systematically integrate genomic, epigenomic, transcriptomic, proteomic, and metabolomic data (271–274). The core value of such integrative analysis lies in its capacity to construct complete pathological regulatory networks spanning from upstream genetic mutations or epigenetic modifications, through alterations in transcription and translation, to final metabolic functional outputs, thereby identifying core driver nodes that decisively influence disease states within complex molecular networks as the most efficient therapeutic targets (275–277). More importantly, through clustering analysis of patient multi-omics data, SAE subtypes based on distinct molecular characteristics may be discovered, laying foundations for stratified treatment and precision medicine (274, 278–280). However, multi-omics integration faces numerous challenges including data standardization, dimensional disparities between different omics datasets, complexity of bioinformatic analysis algorithms, and massive data storage and computational requirements, urgently necessitating close interdisciplinary collaboration (279–282).

### 5.3 Breakthrough potential of single-cell resolution metabolomics

The breakthrough potential of single-cell resolution metabolomics is becoming a highly anticipated frontier direction within the field. Traditional tissue metabolomics analysis provides averaged metabolic states across millions of cells, inevitably masking critical metabolic heterogeneity between different cell types and even among different subpopulations within the same cell type (283–286). Considering that the brain is a complex organ composed of multiple highly specialized cell types with fundamentally distinct metabolic reprogramming patterns in SAE—microglia, neurons, and astrocytes—achieving single-cell resolution metabolic analysis holds paramount significance for understanding SAE pathophysiology (19, 287–289). Although single-cell transcriptomics has been extensively applied in sepsis research and revealed immune cell heterogeneity, single-cell metabolomics remains in its infancy, facing technical bottlenecks including extremely low metabolite content within single cells, extraordinarily high detection sensitivity requirements, and loss of spatial information (290–293). Currently, no published studies have directly applied single-cell metabolomics in the SAE field. With continuous development of technologies including mass spectrometry imaging, microfluidics, and novel probes, single-cell metabolomics is expected to achieve breakthroughs within the coming years (294, 295). Once realized, it will enable precise delineation of specific metabolic atlases for each brain cell type in SAE, discovery of rare but potentially critical cell subpopulations in disease progression, revelation of “metabolic communication” and nutritional support networks between cells through metabolites, and assessment of drug effects on metabolic states of different cell types at the single-cell level (296–298). Single-cell multi-omics, particularly the combination of single-cell transcriptomics with single-cell metabolomics, will provide unprecedented high-resolution perspectives for deconstructing SAE complexity (299, 300).

### 5.4 Four feasible pathways for translational medicine

Four feasible pathways for translational medicine illuminate the direction for ultimate realization of SAE research value. Translating basic research discoveries into clinical practice benefits represents the ultimate objective of SAE research. Based on current research progress, future translational medicine exploration can proceed systematically along four pathways (301, 302). First is clinical validation and application of biomarkers: systematically validating metabolic reprogramming-driven candidate biomarkers discovered in animal models and small cohorts within large multicenter prospective clinical cohorts, developing clinical detection systems applicable for early diagnosis, risk stratification, and prognostic prediction (303–306). Second is drug repurposing strategies: prioritizing randomized controlled clinical trials of metabolic modulators already in clinical use with established safety profiles for SAE, substantially shortening development timelines and reducing costs (307–309). Third is development of novel targeted drugs: designing and developing novel small-molecule inhibitors or activators with high specificity and favorable blood-brain barrier penetrability targeting metabolic enzymes or pathways validated as core driver nodes in basic research (310–313). Fourth is exploration of combination therapeutic strategies: considering the multidimensional nature of SAE pathological mechanisms, future treatment will likely involve not single-target intervention but comprehensive combination regimens, such as combining metabolism-targeting drugs with anti-inflammatory agents or neuroprotectants to achieve synergistic therapeutic effects (86, 191, 198, 314). These four pathways complement one another, collectively constituting a systematic translational framework from laboratory to bedside.

## 6 Conclusion

This review systematically demonstrates that metabolic reprogramming offers an integrative framework for understanding SAE pathophysiology. The four core metabolic pathways—glycolysis/Warburg effect, TCA cycle dysfunction, lipid dysregulation, and amino acid disturbance—interact to drive neuroinflammatory activation, energy crisis, and cellular damage across microglia, neurons, and astrocytes. The gut-brain axis further modulates these processes through microbiota-derived metabolites.

However, significant evidence gaps must be acknowledged. Most mechanistic insights derive from animal models and require validation in human SAE. Causal relationships between specific metabolic alterations and neurological outcomes remain incompletely established. The translational barriers facing metabolic biomarkers and targeted therapies—including assay standardization, therapeutic time windows, and patient heterogeneity—are substantial and have impeded clinical progress.

**Future priorities should include:** (1) longitudinal multi-omics studies capturing metabolic evolution from acute illness through long-term cognitive outcomes; (2) single-cell and spatial metabolomics to resolve cellular heterogeneity; (3) biomarker-guided therapeutic trials testing whether metabolic modulation improves patient-centered outcomes; and (4) rigorous validation of preclinical findings in clinically relevant human cohorts. Addressing these challenges will determine whether metabolic reprogramming can transition from a compelling research framework to a clinically actionable paradigm for SAE prevention and treatment.

However, despite substantial progress achieved through existing experimental findings, we must soberly recognize the deficiencies and limitations present in current research. First, causal relationship verification remains insufficient; the majority of studies, particularly human investigations, are essentially correlational in nature. While we observe that alterations in specific metabolites are closely associated with SAE occurrence and progression, whether such alterations represent driving factors, pathological consequences, or merely accompanying phenomena remains to be rigorously validated through more stringent experimental approaches including gene editing, isotope tracing, and metabolic flux analysis. Second, serious neglect of cellular heterogeneity constrains our in-depth understanding of SAE pathological essence; most existing data derive from bulk tissue analysis, which completely masks critical metabolic differences between different cell types and even among different subpopulations within the same cell type, while our knowledge regarding specific metabolic reprogramming patterns and functional significance of non-immune cells such as astrocytes, oligodendrocytes, and vascular endothelial cells in SAE remains remarkably limited. Third, substantial challenges in clinical translation cannot be overlooked; from young healthy inbred animal models to elderly patients with multiple comorbidities, from standardized experimental conditions to complex and variable clinical scenarios, and from short-term observational indicators to long-term cognitive prognosis, differences at every stage may constitute fatal barriers to translating basic research findings into clinical practice.

Based on profound reflection upon these limitations, future research should seek breakthroughs in the following strategic directions to bring revolutionary advances to SAE diagnosis and treatment. First, embrace single-cell and spatial resolution technologies: vigorously develop and apply cutting-edge methodologies in single-cell metabolomics and spatial metabolomics to precisely delineate, with highest resolution, the metabolic states, metabolic dynamics, spatial distribution patterns, and intercellular interactions of different cell types within SAE lesions, thereby truly deconstructing cellular heterogeneity and molecular network complexity of the disease. Second, conduct longitudinal, multi-omics integrated clinical studies: design large-scale prospective clinical cohorts with continuous collection of multidimensional biological samples from patients at various critical time points throughout disease evolution—from intensive care unit admission through acute phase outcomes to long-term follow-up—for longitudinal integrated analysis of genomics, transcriptomics, proteomics, and metabolomics, thereby capturing dynamic evolution patterns of metabolic reprogramming and discovering functional biomarkers capable of early SAE risk warning, precise disease severity assessment, and prediction of long-term cognitive prognosis. Third, deepen systematic research on the gut-brain-immune metabolic axis: comprehensively utilize metagenomics, metabolomics, and immunological approaches to systematically resolve how intestinal microbiota and their metabolites shape SAE metabolic microenvironments and neurological functional outcomes through modulation of systemic immune states and direct action on the central nervous system, and explore novel “outside-in” intervention strategies based on probiotics, prebiotics, and fecal microbiota transplantation for intestinal microbiota modulation. Fourth, develop precision-targeted therapeutic regimens: relying upon biomarker-driven patient stratification systems combined with single-cell resolution technological approaches, develop novel drugs and intelligent delivery systems capable of specifically targeting key cell types or specific metabolic pathways, achieving precise metabolic modulation of activated microglia, energy-crisis neurons, or functionally transformed astrocytes, ultimately advancing toward a new era of personalized, precision treatment for SAE.

In conclusion, positioning metabolic reprogramming at the core of SAE research not only provides unprecedented theoretical depth and breadth of vision for understanding this complex syndrome but also ignites new hope for overcoming the severe clinical challenges it presents. While the road ahead is undoubtedly fraught with difficulties and obstacles, with the continuous emergence of novel technologies and deepening interdisciplinary collaboration, we have ample reason to believe that a new paradigm for SAE diagnosis and treatment—centered on metabolic modulation, characterized by precision diagnosis, and oriented toward individualized therapy—is gradually taking shape and maturing.
